# A Retrospective Investigation of Thiamin and Energy Intakes Following an Outbreak of Beriberi in the Gambia

**DOI:** 10.3390/nu3010135

**Published:** 2011-01-19

**Authors:** David I. Thurnham, Angela E. Cathcart, Margaret B. E. Livingstone

**Affiliations:** Northern Ireland Centre for Food & Health, University of Ulster, Coleraine BT52 1SA, UK; Email: angelacathcart@yahoo.co.uk (A.E.C.); MBE.Livingstone@ulster.ac.uk (M.B.E.L.)

**Keywords:** thiamin intake, energy intake, seasonality, beriberi

## Abstract

In the early part of the rainy season in 1988, an outbreak of beriberi occurred in free-living adults in a relatively small area in the North Bank region of The Gambia. In 1995 we selected two compounds in a village called Chilla situated within the affected district to retrospectively examine dietary factors potentially contributing to the outbreak. There had previously been cases of beriberi in one compound (BBC) but not in the other (NBC). We measured energy and thiamin intakes for four days on six occasions during the year. We calculated energy and thiamin intakes of people living in the two compounds and foods were collected for thiamin analysis through the year. Thiamin:Energy ratios only met international recommendations in the immediate post‑harvest season when energy and thiamin intakes were highest and then fell through the year. In the rainy season when food was short and labour was heaviest, energy intakes were lower in the NBC but thiamin:energy ratios were lower in BBC. Records of rainfall in 1988 collected near the village indicated that the amount in August was twice the average. We suggest the heavy rainfall may have increased farm workload and reduced income from outside-village work activity. The lower energy intakes in the NBC may have forced adults to rest thus sparing thiamin demands and delaying onset of beriberi. In contrast, the higher energy intake of adults in the BBC may have enabled them to continue working, thus increasing demands for thiamin and inducing the earlier onset of beriberi.

## Abbreviations

BBC: beriberi compound;
NBC: non-beriberi compound;
BMR: basal metabolic rate;
EI: energy intake;
EE: energy expenditure;
FFQ: food frequency questionnaire;
SES: socio-economic status;
MJ: mega-joules;
SP: study period, approximately two months;
PHC: Public health Centre;
WF: weighting factor;
WMI: weighed meal intake method

## 1. Introduction

During the early part of the rainy season of 1988, at least 140 people attended clinics in the North Bank Division of The Gambia with symptoms characteristic of wet beriberi. Beriberi is the clinical disease associated with thiamin deficiency and the symptoms reported by the people included shortness of breath, chest pain, anorexia, epigastric discomfort, swollen ankles and paraesthesia or a tingling sensation in the skin. There were 22 deaths during July and August before the outbreak was brought to a rapid halt by the distribution of thiamin in September throughout the affected area. Investigations by workers immediately after the outbreak suggested that the consumption of imported white rice may have been the main aetiological factor responsible for the attack [1] as imports of highly milled rice grain had risen steadily over the previous 20 years [2], the consumption of white rice in the West African diet has replaced courser and more traditional grains [3] and there has been a long association of rice consumption with beriberi in Asia and other parts of the world [4,5,6,7].

Many of the people who reported symptoms of beriberi came from a village of approximately 1000 people called Chilla. Chilla is in the North Bank Division of The Gambia which is the poorest region in the country. All the villagers were subsistence farmers and had minimal education apart from Koranic teaching to boys and men. The principal crops grown were a staple of pearl millet (*Pennisetum gambiense*) and a cash crop of groundnuts (*Arachis hypogaea*). Both crops require the rain for growth so crop yields were determined by restrictions imposed by available manpower for land preparation. The climate is typically sahelian with a short monomodal rainy season from June to October and a long dry season; November to May. Millet was the main cereal consumed but the diet was supplemented with imported milled rice (*Oryza sativa*). Approximately 6.5% (*n* = 63) of people in the village were affected by sub-acute thiamin deficiency with symptoms of peripheral oedema and mixed motor and sensory neuropathy. The incidence rates were three times more common in men than women and almost one third of the men who developed beriberi were 20–29 years [1]. Thus the condition targeted a group who were presumably the fittest and strongest individuals in the community but nevertheless the total number of people affected in the village was small. Therefore it was of interest to investigate why this group was more vulnerable to developing beriberi.

Many factors are important in the aetiology of beriberi [4] but overall current outbreaks usually occur where people are restricted as in prisons [8,9] or abuse alcohol as it reduces absorption of thiamin [10]. None of these factors was relevant in The Gambia as people were free living and alcohol was not drunk in this Muslim community. Thiamin is needed principally to metabolise carbohydrate through the citric acid cycle and produce energy. Epidemiological evidence suggests beriberi occurs when the intake of thiamin is <0.2 mg/4.2 MJ (1000 kcal) and current requirements are set at 0.4 mg/4.2 MJ [11]. The thiamin concentration of fresh milled rice is reported to be 0.08 mg/100 g [12] which equates to 0.22 mg/4.2 MJ but when boiled the thiamin falls to 0.01 mg/100 g making it inadequate by itself to meet thiamin requirements. Free-living people will normal eat a mixed diet and foods like meat, fish, nuts etc improve the thiamin:energy ratio. However, the outbreak occurred at the time of the year known as the “hungry season” when food reserves were low and much manual labour was needed for ploughing, planting and weeding. Furthermore, any cereal remaining had been stored since the last harvest and nutrient content may have fallen with age and poor storage conditions. The lifestyles of the inhabitants of Chilla were typical of many West African subsistence farmers [13] and beriberi has been reported elsewhere in both East and West Africa [14,15,16]. There are also reports that the acute heart failure of beriberi may often be misdiagnosed even in industrialised countries [17]. Since the risk of beriberi may be high in many communities across this region, we decided it was important to try and quantify thiamin and energy intakes throughout a typical year to determine their possible role in precipitating the outbreak. 

In the following retrospective report we describe studies on energy and thiamin availability carried out from 1 February 1995 for a full calendar year in two family groups; one who had experienced beriberi in some family members in 1988 and the other that was unaffected. In addition, the thiamin content of frequently consumed foods was collected throughout the year and the thiamin content was analysed and reported here.

## 2. Experimental Section

### 2.1. Demography

Before the study started, ethical permission was sought and obtained from the Medical Research Council/Gambian Government Ethical Committee. The purposes of the project were described to the village elders and their permission was sought and obtained for the work to take place. Work in the village was done by AEC with the assistance of a translator and two field workers. A census was done in the village in August 1994 that identified 1027 inhabitants living in 44 compounds ranging in size from 6 to 77 inhabitants. The headman in each compound provided the information on the demographics of his extended families and this identified 33 compounds as Wollof-speaking with five Fula and four Mandinka. The census also found that 24 compounds were affected by beriberi in 1988 comprising 22 Wollof and 2 Fula. Using a socio-economic questionnaire developed by the MRC workers in Keneba, 22 compounds had high socio-economic status (SES), 14 were medium and 8 were poor. The two study compounds selected were both Wollof-speaking and were chosen as both compounds had the largest number of young adult males (Table 1) and both were high SES. The absence of beriberi in 1988 in the NBC selected for the study was independently confirmed by the village health worker.

### 2.2. Study Design

The 12 month study was divided into six study periods (SP), each of approximately two months duration. The following measurements were repeated in each SP; food and nutrient intakes, energy expenditure and morbidity rates (the last two will be reported elsewhere). Results for the first SP for the January–February period are not shown as the studies were regarded as a trial run. 

**Table 1:** Description of inhabitant of the two study compounds in the village of Chilla ^1,2^.

Age range Years	Non-beriberi compound		Beriberi compound	
	Men	Women	Men	Women
<10	8	11	13	11
10–19	5	4	3	5
20–29	1	6	7	3
30–39	4	3	0	4
40–49	2	0	4	1
>50	0	1	2	2
Total	20	1	29	26
15–49	10	10	8	9

^1^ Details on the two compounds were obtained in August 1994. They were selected on the basis of containing a high proportion of men in the age range 15–49 years who were the group with greatest vulnerability to beriberi in 1988; ^2^ The compound names indicate whether symptoms of beriberi occurred (BBC) or did not occur (NBC) in 1988.

### 2.3. Food and Nutrient Intake

The techniques (weighed-meal-intake method, WMI) used in this work were developed by Hudson [18] to cope with situations where people ate from communal bowls of food. Detailed observations and measurements of meal preparation were made and analysed using food composition tables to calculate the nutrient content of each meal. The distribution of food between individuals was then estimated by a suitable algorithm. Previous work using the, doubly-labelled water technique established that the energy intake calculated from detailed observation of two cooked meals per day was equivalent to approximately 80% of the total energy expenditure and, by inference, total dietary energy intake [18]. The remaining unaccounted energy intake is likely to have been obtained from uncooked “snack foods”, such as groundnuts, raw fruit and vegetables, or from cooked food obtained, by purchase or as a gift, away from the home. 

The WMI method was carried out for four days in each study period in the two study compounds and to determine possible sources of the unexplained energy intake a food frequency questionnaire was used to determine patterns of snacking by individual study subjects. Cooking was shared by the women in a compound and the cooked food was distributed and eaten in feeding groups so the evening before each data collection, the compound was visited to ascertain who was cooking on the study days. The last minute notice of the visit was to try to prevent any alterations in usual dietary patterns for example by purchasing of special foods to impress the observer. For the WMI methods the following information was collected: (1) identity and weight of the ingredients used in the cooking; (2) weight of the prepared foods added to each bowl for feeding group consumption; and (3) calculation of nutrient composition of the bowl’s food content; (4) records of names, sex, age and weights of each person eating from the feeding group bowl. At the end of each four day food observation period, all persons appearing in the food intake records, if still present in the village, were weighed fasting and barefoot to the nearest 0.5 kg using a portable scale (CMS Weighing Equipment, Camden, London, UK). 

The nutrient composition of all foods appearing on the observed WMI records was calculated using a computerised dietary analysis program (Comp-eat, Lifeline, Nutritional Services Ltd., London, UK). Foods not present in the database were added where necessary using information from a variety of sources [19,20,21]. Seasonal differences in the thiamin content of specific foods were also incorporated into the database. Calculating the weighting factors (WF) to apportion the proportion of food and nutrients consumed at a meal used the following algorithm that recognises the non-proportional relationship between energy requirements and body weight [18]. 

      WF factor = ^int^[(*Wt* − 10.5)/10] × 0.25 + 1.5

where Wt is body weight (kg) for people weighing >10.5 kg for whom WFs were calculated using the algorithm but subjects weighing <10.5 kg were assigned a WF of 1.0. The sum of the WFs of all people eating from a food bowl was equated to the total nutrients in the bowl and the magnitude of a person’s WF determined the proportion of the nutrients consumed by that individual.

### 2.4. Food Frequency Questionnaire (FFQ)

The FFQ sought information on foods consumed outside the communal eating occasions and was completed by a number of subjects aged 15 to 49 years in the study compounds. The foods included in the FFQ was based on observations and enquiries from local people and included foods eaten all the year round, e.g., cereals, meats, groundnuts, green tea and cola nuts and foods that were only seasonally available like fruits and bush foods. Each subject present at the end of the SP was asked to recall how many times he/she had consumed the listed foods in the previous 4 weeks. Results were expressed as the mean frequency of consumption over the four week period.

Basal metabolic rate: The BMR was calculated from the predictive equations based on weight and height [22] that were measured on all subjects in each SP. Mean daily energy intakes (EI) of each subject group were compared to the mean basal metabolic rate (BMR) of that group (EI/BMR) to provide indices of adequacy of the energy intakes. 

### 2.5. Thiamin Analysis of Foods Eaten in Chilla

Food collection and storage: Millet products namely the raw materials the fine flour and the grain and steamed products prepared by the “cherreh” and “nyelling” processes were collected without prior notice from different compounds from those in the study approximately every six weeks from January to November. Samples of raw rice (*n* = 14), boiled rice (*n* = 14), raw groundnuts (*n* = 28), roasted groundnuts (*n* = 6) and groundnut paste (*n* = 21), green leaves (*n* = 7), bush foods (*n* = 9) and other miscellaneous items (*n* = 17) were collected at various points throughout the year. Samples were collected in freezer bags and stored for approximately four days in the freezer compartment of a domestic gas fridge (<4 °C). They were then transported to the MRC laboratories in Fajara where they were stored at −20 °C for one to 12 months. In January 1996 they were shipped in dry ice to the University of Ulster and stored at −60 °C for approximately 8 months in preparation for thiamin analysis. Frozen or dehydrated foods generally incur little loss of thiamin on storage [23].

### 2.6. Analysis of Thiamin

The technique used was that of Bailey and Finglas [21]. Food samples were thawed and thoroughly mixed or homogenised. Duplicate 5 g portions of the foods and water blanks were weighed into 100 mL conical flasks and then heated for 20 min at 100 °C with 25 mL 0.1 M-hydrochloric acid (SS40-D Grant water-bath, Cambridge Instruments Ltd.). After cooling to 37 °C, 5 mL freshly prepared enzyme preparation was added (1% w/v taka-diastase/2% w/v sodium hydroxide in 2 M-sodium acetate buffer adjusted to pH 4.5 with 15% w/v NaOH). After adjusting the pH to 4.5 using 3.75 M-NaOH, the flasks were left overnight (~12 h) in a shaking water-bath at 37 °C. After cooling each solution was made up to 50 mL with 0.1 M-HCL, filtered through Whatman 541 filter paper and aliquots transferred to polythene vials (2 mL, LIP Equipment & Services Ltd., H & L Suppliers Ltd., Belfast, UK) and stored at −60 °C until analysis.

Duplicate samples (1 mL) were pipetted into 4mL screw-topped, amber glass vials (Pierce & Warriner Ltd., UK) containing 100 µL 0.1 M-HCl. Using 1 mL of the blank solutions, standards were prepared by adding 100 µL 0.5–2.0 mg/L thiamin hydrochloride. Fresh K_3_Fe(CN)_6_ oxidising reagent (1 mL) was added to each sample and standard and mixed by inversion. Isobutanol (1.5 mL) was then added to all tubes, the samples were shaken vigorously for 2 min and then centrifuged at 2000 rpm for 10 min. The upper isobutanol layer was removed into autosampler vials for liquid chromatography. Samples were injected onto a 5 µm 25 × 0.4 cm Lichrosorb Li 60 column (Merck Hichrom Ltd., Reading) and eluted isocratically with a mobile phase of chloroform-methanol (80–20 v/v). Thiamin was measured using a fluorescence detector (model LC1255 Spectrochrom Ltd., Brackley, Northants, UK) and excitation and emission wavelengths of 375 and 430 nm respectively. Concentrations of thiamin were calculated using the standard curve generated with each batch of samples. Samples were repeated if duplicate variation was >10%. Intra-assay variation based on duplicates was 4.4% and inter-assay variation was 20%.

### 2.7. Statistics

Statistics were done using the Statistical Package for Social Sciences (SPSS Inc., Chicago, U.S.) version 6 for Windows. For the WMI analyses, data were log transformed before analysis and differences between compounds, genders and study periods compared using one way analysis of variance and significant differences between groups assessed using the least significant differences (LSD) test. FFQ analyses were done using non-parametric tests. Study periods were identified when a particular food was not available for consumption and these were excluded from analysis. Seasonal and subject group differences were compared using Kruskall-Wallis one way analysis of variance and significant differences between groups assessed by the Mann-Whitney U-Wilcoxan Rank Sum W Test.

## 3. Results

Inhabitants within the compounds separated into feeding groups to eat their meals. In the NBC there were six feeding groups comprising: married men, the head man and young sons (*n* = 5), unmarried men (*n* = 7), old women and unmarried women (*n* = 9), married women and their infants (*n* = 8) and a small family unit (*n* = 5). In the BBC there were four feeding groups: the headman and all male children (*n* = 8), adult men, married and single (*n* = 10), older women and all female children (*n* = 7) and adult females and infants (*n* = 10). Food intake was measured for each feeding group on four successive days within the five SP from March through to December in 1995. Assuming adequate food availability, three cooked meals were consumed in the compounds daily; breakfast around 8:00 a.m., lunch around 2:00 p.m. and dinner around 8:00 p.m. Meals generally consisted of cereal (rice or millet but usually millet), sauce and added meat, fish or vegetables. The sauce was ground nut or oil based. Boiled rice or “nyelling” millet was eaten for lunch while “cherreh” millet was only eaten at dinner. Food was cooked freshly for lunch and dinner while breakfast was usually some of the previous night’s dinner. During the late farming season (September) when men worked in the fields from day break (6:30 a.m.), breakfast was sometimes taken to the fields.

Energy intakes for the occupants of the two compounds separately were calculated as MJ/WF/day (Table 2) and showed some evidence of a fall during the early rainy season. However, the intake was only significantly lower in the second half of the rainy season and only in NBC. Similar results were obtained for the adult (15–49 years) men and women separately (Table 3). Daily intakes of thiamin (mg/4.2 MJ) in the rainy season showed a similar picture to energy intakes but the intake was only significantly lower in the BBC (Table 2). In general the thiamin:energy intake ratios were not different between the genders and only in the post-harvest season (November/December) did men consume a higher ratio in both compounds (Table 3).

**Table 2:** Seasonal variations in energy (MJ/WF/day) and thiamin (mg/4.2 MJ) intakes in the study compounds NBC and BBC through one year ^1,2,3^.

Study period	Weather	Energy (MJ/WF/day)		Thiamin (mg/4.2 MJ)	
		NBC	BBC	NBC	BBC
March/April	Dry	3.0 (0.2) ^a^	3.1 (0.5)	0.29 (0.05) ^a,b^	0.25 (0.04) ^a^
May/June	Dry	3.0 (0.1) ^a,b^	3.1 (0.3)	0.22 (0.05) ^a,b^	0.22 (0.08) ^a^
July/August	Wet	2.5 (0.1) ^c^	2.9 (0.3)	0.37 (0.30) ^a^	0.12 (0.04) ^b^
September/October	Wet	1.4 (0.1) ^d,x^	3.0 (0.1) ^y^	0.16 (0.01) ^b,x^	0.09 (0.02) ^b,y^
November/December	Dry	3.3 (0.1) ^b^	3.3 (0.8	0.62 (0.05) ^b^	0.53 (0.07) ^c^
ANOVA; *P*-value		0.001	0.791	0.003	<0.001
Yearly average		2.7 (0.7)	3.1 (0.4)	0.33 (0.21)	0.25 (0.17)

^1^ Values were geometric mean intakes (SD) for four days observation except in BBC in September/October when *n* = 3. WF was the unit of measurement by which all subjects were apportioned their intake from the communal feeding bowl; ^2^ NBC designates a compound with no beriberi in 1988 while BBC had cases of beriberi in that year; ^3^ Data were log transformed before repeated measures analysis of variance (ANOVA) and subsequent group tests; seasonal differences ^(a,b,c)^ and compound differences (^x,y^) are shown where mean values do not share a common superscript letter (*P* < 0.05, LSD test). Differences between compounds within study periods are shown by unlike superscripts (^x,y^; independent *t*-tests).

Measured energy intakes (EI) from food were expressed as a multiple of the respective predicted BMRs for all study subjects 15–49 years and geometric mean EI/BMRs were calculated to determine the adequacy of the energy intakes (Table 4). Subjects in the NBC showed the lowest ratios with the average value for the whole year being only 10% above the BMR. During the wet SP, EI/BMR were <1.0, *i.e.*, subjects were in negative energy balance and not consuming enough energy to sustain normal physiological functions.

**Table 3:** Seasonal and gender differences in intake of energy and thiamin through the year in the study compounds ^1,2^.

Study period	Energy Intake (MJ/WF/day) mean (SD)				Thiamin intake (mg/4.2 MJ) mean (SD)			
	NBC		BBC		NBC		BBC	
	Men	Women	Men	Women	Men	Women	Men	Women
2 (dry)	3.0 ^a^ (0.4)	2.6 ^a,b^ (0.2)	2.9 (0.2)	3.3 (0.9)	0.27 ^a^ (0.04)	0.28 ^a,b^ (0.05)	0.22 ^a^ (0.09)	0.27 ^a^ (0.07)
3 (dry)	3.0 ^a^ (0.2)	3.5 ^d^ (0.3)	3.2 (0.3)	3.3 (0.4)	0.19 ^a^ (0.04)	0.24 ^a,b^ (0.09)	0.20 ^a,b^ (0.07)	0.23 ^a^ (0.09)
4 (wet)	2.8 ^a,#^ (0.2)	2.1 ^a,c^ (0.2)	3.3 ^#^ (0.5)	2.5 (0.3)	0.24 ^a^ (0.24)	0.41 ^a,c^ (0.24)	0.11 ^a,b^ (0.04)	0.13 ^b^ (0.05)
5 (wet)	1.4 ^b,xx^ (0.3)	1.6 ^c^ (0.1)	3.5 ^#^ (0.1)	3.1 (0.3)	0.16 ^a,xx^ (0.01)	0.15 ^b^ (0.01)	0.08 ^b^ (0.01)	0.09 ^b^ (0.02)
6 (dry)	4.1 ^c^ (0.1)	2.7 ^b^ (0.7)	3.7 (0.6)	3.4 (1.0)	0.67 ^b,#^ (0.05)	0.51 ^c^ (0.01)	0.58 ^c,#^ (0.14)	0.52 ^a,c^ (0.07)
ANOVA *P*-value	0.001	0.001	0.145	0.355	<0.001	0.005	<0.001	<0.001
Yearly average	2.9 ^#^ (0.9)	2.5 (0.7)	3.3 (0.4)	3.1 (0.7)	0.31 (0.22)	0.32 (0.16)	0.25 (0.20)	0.25 (0.17)

^1^ Values were mean intakes (SD) for 4 days observation except in BBC in September/October when *n* = 3. WF was the unit of measurement by which all subjects were apportioned their intake from the communal feeding bowl. For other information on compounds see Table 1; ^2^ Values shown are geometric means (SD) for men and women 15 to 49 years in the respective compounds (see Table 1); ^3^ Data were log transformed before repeated measures ANOVA and subsequent group tests; seasonal differences are shown where mean values do not share a common superscript letter ^(a,b,c)^ (*P* < 0.05, LSD test); ^#^ Gender differences found within the compound (*P* < 0.05, LSD test); ^xx^ Differences between compounds (*P* < 0.05, LSD test).

**Table 4:** Seasonal and gender variations in energy intakes in relation to basal metabolic rates (EI:BMR) of men and women in the study compounds ^1,2,3^.

Months	NBC		BBC		ANOVA *P*-vaule	All subjects
	Men	Women	Men	Women		
March, April	1.13 ^a,x^ (0.17) *n* = 7	1.21 ^a,x^ (0.10) *n* = 7	1.15 ^a,b,x^ (0.10) *n* = 7	1.42 ^a,y^ (0.19) *n* = 9	0.001	1.24 ^a^ (0.18) *n* = 30
May, June	1.10 ^a,x^ (0.28) *n* = 7	1.48 ^b,y^ (0.15) *n* = 8	1.11 ^a,x^ (0.13) *n* = 7	1.37 ^a,y^ (0.12) *n* = 8	<0.001	1.28 ^a^ (0.24) *n* = 30
July, August	1.01 ^a,x^ (0.14) *n* = 6	0.93 ^c,x^ (0.07) *n* = 8	1.26 ^b,c,y^ (0.05) *n* = 8	1.10 ^b,x^ (0.23) *n* = 8	<0.001	1.08 ^b^ (0.19) *n* = 30
September, October	0.52 ^b,x^ (0.05) *n* = 4	0.62 ^d,x^ (0.09) *n* = 8	1.33 ^c,y^ (0.16) *n* = 6	1.24 ^a,b,y^ (0.23) *n* = 8	<0.001	0.96 ^b^ (0.38) *n* = 26
November, December	1.40 ^c^ (0.19) *n* = 6	1.16 ^a^ (0.18) *n* = 8	1.34 ^c^ (0.13) *n* = 8	1.39 ^a^ (0.26) *n* = 8	0.086	1.32 ^c^ (0.21) *n* = 30
ANOVA *P*-value	<0.001	<0.001	0.001	0.024		<0.001
All months	1.07 (0.31) *n* = 30	1.09 (0.31) *n* = 41	1.24 (0.14) *n* = 36	1.31 (0.27) *n* = 41	<0.001	

^1^ Data are geometric means (SD) of energy intakes (MJ/day) for individuals in feeding groups multiplied by their respective WF and divided by their respective BMRs. For information on study compounds see Table 1; ^2^ Food intake measurements are those described in Table 2; ^3^ Data were log transformed before repeated measures ANOVA and subsequent group tests; seasonal differences are shown where mean values do not share a common superscript letter ^(a,b,c)^(*P* < 0.05, LSD test). Similar methods were used to determine gender and compound differences (^x,y^).

The thiamin content of the steamed millet is shown in Table 5 together with the months of collection. Millet is harvested in late September so the October sample was the freshest one collected. By March the concentration of thiamin had halved and in the following August and September only 10–20% of the original content remained. Of the snack items, fruits, bush foods and green leaves analysed (mean ± SD/100 g), only raw groundnuts (0.58 ± 0.15 mg/100 g) and okra flour (0.18 ± 0.02) contained relatively large amounts of thiamin. Imported raw rice (0.08 ± 0.06) had lost almost all its thiamin after cooking (0.01 ± 0.01). Roasted groundnuts and groundnut paste (0.06 ± 0.01 and 0.05 ± 0.01, respectively) contained significant amounts of thiamin and as they were added to the main meals as sauces, they were incorporated in the food composition database to calculate intakes.

**Table 5:** The thiamin content (mg/100 g) of the steamed millet products prepared in the village for their main meals ^1^.

Collection month	Steamed millet products mg thiamin/100 g			
	Number of samples	Cherreh	Number of samples	Nyelling
March	5	0.063 (0.072)	3	0.032 (0.011)
May	4	0.032 (0.039)	3	0.073 (0.021)
August	3	0.018 (0.005)	3	0.006 (0.007)
September	5	0.011 (0.016)	4	0.011 (0.009)
October	5	0.132 (0.028)	2	0.060 (0.006)

^1^ Values are means ± standard deviations.

Semi-quantitative information obtained from the FFQ is presented in Table 6 where figures represent the number of times the most frequently-consumed foods were eaten in a four-week recall period by the adults in the two study compounds. Aubergine, bitter tomato, cassava and banana were grown in the village gardens and were eaten in all study periods. Mangoes were abundant and free for everybody in late April through to June and in the May/June study period only one of the 27 adults had not eaten mango during the recall period. Snacking on groundnuts was a daily or every-other-day occurrence throughout the year in more than 90% of persons questioned. However during the rainy season (SP 4 & 5) groundnut stocks had dwindled and intake was much less frequent. It was not possible to quantify either energy or thiamin intakes from these data.

**Figure 1:**
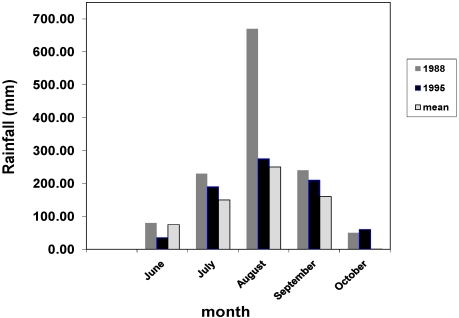
Monthly rainfall (mm) at Kerewan in 1988, 1995 and the average for the period 1949 to 1995.

As indicated elsewhere the villagers were subsistence farmers their eating and work activities were very much determined by the seasonal calendar. Table 7 shows the typical work activities observed in 1995 and these are undoubtedly characteristic of every year. What the table cannot illustrate however is the differences in the intensities of effort that are determined by changes in the weather patterns in different years. One of the factors mentioned by Tang and colleagues [1] when they first reported the outbreak in 1988 was that the rainy season that year was especially wet and that the rainfall in the area of Chilla was especially heavy. Figure 1 shows rainfall statistics for 1988, 1995 and the mean figure for data collected since records began in 1949 [24]. There are six meteorological stations in The Gambia and Kerewan is located closest to Chilla. The figure shows the rainfall was heaviest most years in August but in 1988 it was almost double the level in other years. Three stations recorded above average rainfall in 1988, Banjul (1000 mm), Yundum (1250 mm) and Kerewan, but the total years rainfall recorded in Kerewan was highest at 1311 mm. 

**Table 6:** Seasons of most frequent consumption of snack foods and individual fruits, vegetables and bush foods eaten in Chilla ^1,2,3^.

Food *Latin name*		Months of most frequent consumption	Number reporting consumption	SP when NOT available	Thiamin content (mg/100 g)
**Vegetables**					
Aubergine *Solanum melongena*		September/October	13	0 *	0.05
Bitter tomato *Solanum incanum*		March/April	9	0	0.11 **
Cabbage *Brassica oleracea*		March/April	4	4, 5, 6	0.03–0.06
Cassava *Manihot utilissima*		September/October	5	0	0.005 [16]
Maize *Zea mays*		September/October	6	2, 3, 6	0.20
Pumpkin *Curcubita* spp.		November/December	5	3, 4	0.04
Sweet potato *Ipomoea batatas*		March to June & November/December	>1	5	0.08
**Fruits**					
Baobab fruit *Adansonia digitata*		March/April	1	5, 6	NA
Banana *Musa* spp.		March/April	4	0	0.04
Guava *Ugni molinae*		July to December	1	2, 3	0.04
Mango *Mangifera indica*		May/June	12	6	0.03
Orange *Citrus sinensis*		March to June September to December	2	4	0.10
			>1		
Papaya *Carica papaya*		March/April	3	4	0.02
Custard apple *Annona seneaglaensis*		September/October	>1	2, 3, 4, 6	NA
Water melon *Passiflora laurifolia*		November/December	3	2, 3, 4, 5	0.02
**Bush Foods**					
Bush mango Irvingia gabonensis	Cooked	May/June	11	2, 5 6	NA
	Raw	July/August	4	2, 5 6	0.04 **
Wild dates *Phoenix reclinata*		May/June	>1	2, 4, 5, 6,	0 **
Locust bean pod powder *Ceratonia siliqua*		May/June	5	2, 5, 6	0.4 ** (fresh)
Wild kola nut *Cola acuminata*		July/August	1	2, 3, 5, 6	0 **
**Common snack food**			% reporting consumption	Less frequently available	
Groundnuts (dried, boiled and roasted) *Arachis hypogaea*		March–June, November & December	>90%	5 < 4	0.23–0.9

^1^ Data were collected from adults aged 15–49 years living in the two study compounds and represent mean number of times a food was consumed over a four week period; * a zero indicates a food was always available; ^2^ The numbers of adults interviewed in the study periods (SP) were 31 in SP2 (March April), 27 in SP3 (May June), 28 in SP4 (July August), 26 in SP5 (September October) and 29 in SP6 (November December); ^3^ Values for thiamin content taken from [12], except ** which were samples collected in Chilla and analysed by AEC. NA is not available.

**Table 7:** Divisions of labour by gender during farming and non-farming seasons.

Month of observation	Activities of men	Activities of women in addition to household chores *
January, February	Threshing, bagging and marketing of groundnuts; Collecting firewood from the bush; construction houses latrines & fences; Leisure: playing cards, Koranic recitation, drinking green tea	Shelling ground nuts; cutting firewood in the bush; Leisure‑plaiting hair, drinking green tea
March, April	Cutting sticks in mangrove swamps and bush; making mud bricks; Construction-houses, roofing; honey collection in the bush; Leisure-playing cards, football (every evening), drinking green tea	Gleaming ground nut fields; shelling ground nuts; Leisure-plaiting hair, drinking green tea
May, June	Clearing land, raking grasses; Making mud bricks; Construction-house, roofing; cutting sticks in the bush; shelling groundnuts for sowing; sowing ground nuts begins; Leisure-playing cards, football (every evening), drinking green tea	Firewood collection in the bush; shelling ground nuts; collection of “bush mango” for cooking; Leisure-drinking green tea
July, August	All day sowing and ploughing ground nuts and millet pre-breakfast start; Hoeing ground nuts and millet; Leisure-playing cards, football (once weekly), drinking green tea	Weeding to prepare vegetable gardens in farms or compounds; Hoeing in the ground nut and millet fields; Leisure-drinking green tea
September, October	Some ploughing (a.m. activity only); Harvesting millet and transporting by cart into compound; cutting sticks in the bush; fencing using millet sticks; Weeding field in preparation for watermelon planting; Leisure-drinking green tea	Hoeing in the groundnut and millet fields; collecting leaves from the bush/farm; Harvesting mille‑standing and cutting‑usually a.m. only; with the arrival of fresh millet, pounding appeared to increase; Leisure-drinking green tea
November, December	Harvesting groundnuts using a plough (usually a.m. only); Lifting, windrowing and stacking harvested groundnut plants; Leisure-drinking green tea	Windrowing and stacking harvested groundnut plants (a.m. and p.m.); Leisure‑drinking green tea

* All year round activities of women also included cooking; preparation of whole grain millet; fetching water from the well; sweeping the compound and home; child care; laundry and ironing.

## 4. Discussion

From the information collected in 1995, we estimated that at the start of the rainy season the intake of thiamin relative to energy (0.12–0.37 mg/4.2 MJ), was significantly lower than that measured post harvest (November/December; 0.5–0.6 mg/4.2 MJ) and that even during the dry season, mean thiamin:energy ratios were only a half those measured immediately post-harvest. Previous work in Keneba in The Gambia showed a similar picture in that thiamin intakes of lactating women in November when cereal was abundant were higher (0.8 mg/4.2 MJ) than those in the rainy season (0.58 mg/4.2 MJ) [25]. However, the intakes in Keneba were all higher than those measured in Chilla but no account was taken in the Keneba study of possible thiamin losses in locally-stored cereals. The lower thiamin intakes observed in Keneba in the rainy season were the result of insufficient food at that time of year but at all times in the year the thiamin intake compared favourably with recommended levels of 0.4 mg/4.2 MJ [11,26]. In contrast the thiamin intake in Chilla was only above the recommended value immediately post harvest. 

One food which could not be taken into account fully was the thiamin-rich snack, peanuts, and thiamin intakes during SP 2 and 3 would have been higher than we recorded. However, during SP 4 and 5, the time when beriberi appeared in 1988, we observed little or no peanut consumption. We would not expect peanut supply to have been any different in 1988 compared with 1995, since all foods were limited at the beginning and all through the rainy season [13,27]. 

One reason why our thiamin measurements were lower than those in Keneba was because we incorporated our measurements of local foods into our food composition database. For many of the foods eaten in Chilla, the thiamin content was not known therefore samples were collected throughout the year and especially the predominant staple which was kept locally in thatch-covered stores. Analyses showed considerable deterioration in the thiamin content of the millet samples; an important fact that would have been overlooked had we relied on published values. One additional item appeared in the meals in the NBC during July/August. A corn soy flour blend nutritional supplement was provided to Public Health Centres (PHC) by Catholic Relief Services and the Gambia Food and Nutrition Association. An adult from the NBC worked at the local PHC and help to distribute the supplement, ostensibly for pregnant and lactating mothers, but the supplement was observed by AEC to be consumed by all members of the NBC household. The supplement contained 0.72 mg thiamin/4.2 MJ [28] and may explain why the fall in thiamin intake in the NBC was less pronounced by comparison with that in the BBC. Energy intakes in both compounds were comparable. It was also noted in that SP that no millet was eaten in the NBC and the cereal eaten was rice. That is money was needed to buy the rice which may have restricted food intake at other times in the SP and provided no added benefits in thiamin intake since the content was no higher than that of millet at that time of year.

The thiamin content of imported rice that was available and cooked in the compound displayed the amounts reported in standard food composition tables [12]. That is the samples did not show evidence of the seasonal deterioration in thiamin content that was found in the millet. Presumably rice was stored dry and in relatively good conditions by importers and only brought to Chilla when needed. In contrast millet was stored locally under conditions where temperature and humidity fluctuated throughout the year and this may have contributed to the large loss of thiamin. 

Knowledge of thiamin requirements in energy metabolism is based on studies done when subjects were in energy balance [26]. During negative energy balance the associated tissue catabolism liberates thiamin and some of this may be reutilized [29]. The EI:BMR ratios indicate the adequacy or otherwise of the energy intake of the adults living in Chilla. Accordingly the determination of the energy requirement is essential to fully understand how “the energy from food will balance energy expenditure (EE) [22]”. Estimates of EE by men and women in various seasons will be reported elsewhere. The EI:BMR ratios reported here indicated that energy intakes were insufficient to support even the basal metabolic requirements during the late rainy season in the NBC. Insufficient energy to support the BMR would cause a loss of weight and force adults to rest. Even those subjects whose mean EI:BMR was above 1 are also likely to have been in some degree of negative energy balance. Weight loss would reduce BMR but more importantly would release thiamin from tissues and help to balance energy intake from food. The enforced rest, which was reported by some adults anecdotally, may have spared subjects in the NBC from beriberi.

Hudson [18] reported that energy intakes estimated by his algorithm accounted for approximately 80% of energy requirements estimated from estimates of energy expenditure by the doubly-labelled water technique. The FFQ indicated that a number of snack foods were taken between meals and especially groundnuts which is an important source of both energy and thiamin. No attempt was made to correct food intake data from the information from the FFQ. However from the seasonal availability of foods it would appear the 20% underestimate in energy intake from the WMI [18] was probably obtained by snacking during most of the year except the rainy season, SP 4 & 5, when snack foods were limited. Thus the EI:BMR ratios would be increased overall but they would still remain below 1.0 in the NBC during the late rainy season and indicating a more severe energy depletion relative to the BBC.

No outbreak of beriberi occurred during 1995. However there was a key difference between 1995 and 1988, namely the rainfall. During the rainy season the usual pattern is for rain to be heavy but intermittent; so there would be days of strong sunshine separated by days of heavy rain. Anecdotal reports of the rainfall in 1988 suggested that the rain was continuous and this had important impacts on farming activities. Weeding and hoeing were predominantly female activities during the rainy season in 1995 (Table 7). The weeding method relies on there being sunny days between the rains when cut weeds dry out and die under the hot sun. When the rain was continuous the weeds re-root and weeding had to be repeated. Thus weeding became more demanding in 1988 and men were asked to assist. 

Men were certainly observed to contribute to the farming and compound activities during the year but it was common once ploughing the land and crop sowing was completed, that men sought external work in other towns and villages to supplement the household income. If men were prevented from leaving the compound during the rainy season in 1988 then no extra income could be earned to supplement the needs of the household and food resources would be stretched ever more thinly.

However the demands on human resources imposed by the weather presumably applied equally to both compounds and surprisingly the data collected suggested that nutrition was poorer in the NBC. In the hungry season, the availability of cereals to supply energy was greater in the BBC than the NBC. Thus persons in the BBC may have had the necessary energy to maintain the additional work output to cope with the additional needs imposed by the heavy rain. However a dependency on cereals in the hungry season imposed additional needs for thiamin that were probably not met from the poor quality of cereal at that time of year. From the data in 1995, thiamin:energy ratios were totally inadequate to maintain an efficient energy supply for persons in the BBC during the hungry season and this may be the reason why people in this compound suffered from acute beriberi in 1988. Thiamin:Energy ratios also fell to inadequate levels in the NBC in the second half of the rainy season but no beriberi occurred in this compound in 1988. There are at least two possible reasons for this. There was a mass distribution of thiamin tablets in September of 1988 [1] which may have spared people living in compounds like the NBC but also the poverty and lower energy intakes of people in compounds like the NBC may have forced people to rest sooner so preventing the acute signs of beriberi.

There are number of limitations to this study notably the 7 year gap between the outbreak and this study. However there was no evidence that there had been any financial, nutritional, social or agricultural changes over the period. We used a formula based on a body weight to assess nutrient intakes from the communal feeding bowls that underestimated energy and probably thiamin intakes. In addition we could not assess the nutrient intakes from snacks and as peanuts were an important snack food, this may have underestimated the thiamin intake.

However, on the basis of our estimates, thiamin intakes for the people in Chilla were only really adequate immediately post harvest. Poor storage facilities for the indigenous cereal, millet, may have contributed to the decline in thiamin content which became dangerously low for everybody in the hungry season. The distinguishing feature that may have contributed to beriberi was the ability to maintain EI above the BMR. In the demanding conditions of 1988, the ability to maintain adequate energy intakes in the face of dwindling thiamin supplies may have been the important factor in precipitating acute beriberi. No further cases of beriberi are known to have occurred since 1988 but very little has changed in the village since that time. We believe that to prevent the situation happening again, better storage facilities for the staple cereal are needed. For the farmers this would be a major capital outlay. However, without financial help, further outbreaks in this or other villages in the region appear inevitable if the circumstances are repeated.
